# Three-Dimensional Optical Imaging of Internal Deformations in Polymeric Microscale Mechanical Metamaterials

**DOI:** 10.1021/acs.nanolett.3c04421

**Published:** 2024-01-26

**Authors:** Brian W. Blankenship, Timon Meier, Naichen Zhao, Stefanos Mavrikos, Sophia Arvin, Natalia De La Torre, Brian Hsu, Nathan Seymour, Costas P. Grigoropoulos

**Affiliations:** † Laser Thermal Laboratory, Department of Mechanical Engineering, University of California, Berkeley, California 94720, United States

**Keywords:** mechanical metamaterials, two-photon polymerization, confocal microscopy, polymers

## Abstract

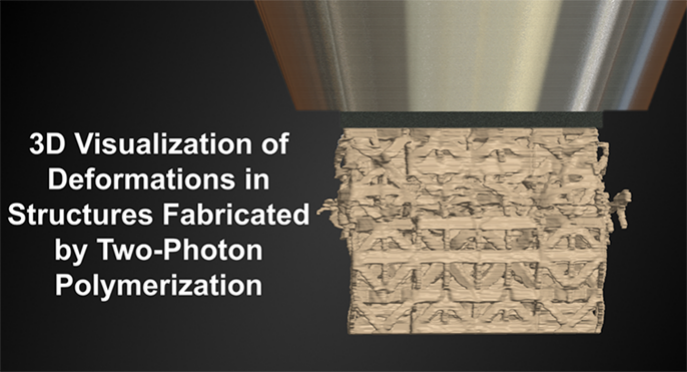

Recent advances in two-photon polymerization fabrication processes are paving the way to creating macroscopic metamaterials with microscale architectures, which exhibit mechanical properties superior to their bulk material counterparts. These metamaterials typically feature lightweight, complex patterns such as lattice or minimal surface structures. Conventional tools for investigating these microscale structures, such as scanning electron microscopy, cannot easily probe the internal features of these structures, which are critical for a comprehensive assessment of their mechanical behavior. In turn, we demonstrate an optical confocal microscopy-based approach that allows for high-resolution optical imaging of internal deformations and fracture processes in microscale metamaterials under mechanical load. We validate this technique by investigating an exemplary metamaterial lattice structure of 80 × 80 × 80 μm^3^ in size. This technique can be extended to other metamaterial systems and holds significant promise to enhance our understanding of their real-world performance under loading conditions.

The application of machine learning and automated routines for geometry generation and mechanical analysis are leading to the rapid exploration of the possible design spaces of mechanical metamaterials with tailored properties.^1−5^ Typically, these materials contain repetitive patterns of low-density unit cells composed of lattices or minimal surface geometries.^6,7^ Through precise manipulation of features at the micro- and nanoscale, these metamaterials can be engineered to exhibit enhanced or even novel properties, surpassing those of traditional bulk materials. So far, these innovative design approaches have yielded materials with extraordinary characteristics, including ultrastiffness,^8,9^ auxetic behavior,^10−12^ negative thermal expansion coefficients,^13−15^ exceptional energy absorption,^3,16^ and atypical material behavior such as “Cauchy symmetry” and roton-like acoustic dispersion among many others.^4,17^ The extent of possible properties has yet to be fully explored, and there are new frontiers in designing materials that possess multiple desired properties.^18^

Coinciding with design advancements has been the development of mesoscale additive manufacturing techniques, enabling the production of arbitrarily shaped metamaterials with intricate nanoscale features and dimensions extending to several centimeters.^19−21^ Among these, two-photon polymerization (TPP) stands out for its fine and versatile printing capabilities, achieving subdiffraction limit feature sizes at volumetric printing rates exceeding 1,000,000 μm^3^/s.^19^ Further enhancements in spatial light modulation and materials science are anticipated to elevate these rates even more, promising a leap in scalability and functionality.^22^

However, as these developments proceed, new diagnostic tools and imaging techniques become necessary to provide advanced *in situ* diagnostics and to better quantify the detailed behavior and performance of these materials. In this context, for instance, microscale metamaterial structures have been functionalized with nanodiamonds containing NV^–^ centers to provide fine temperature and magnetic field measurement capabilities inside of these structures.^23^ Likewise, the functionalization of structures has been explored with other particles such as quantum dots and gold nanoparticles which can impart sensing capabilities.^24^ In terms of imaging, existing methods like scanning electron microscopy (SEM) or helium ion microscopy (HIM) provide detailed surface images that can adequately resolve the nanoscale features of these materials.^25^ However, these imaging modalities fail to capture the internal deformation mechanics of these materials as they rely on the scattering of electrons or ions from the surface of complex structures, which shield the interior members. Thus, subsurface unit cells and interior-facing sections of unit cells on the surface of a larger array are difficult, if not impossible, to image using these techniques.

Alternative techniques utilizing X-ray tomography have been developed that capture the interior deformation of these materials during mechanical compression, yet these techniques require specialized equipment paired to synchrotron radiation sources to achieve resolutions below 500 nm.^26^ Confocal imaging techniques have been recorded across literature to statically image three-dimensional microscale structures made with TPP. However, they do not appear to have been applied in studies involving *in situ* mechanical compression.^27,28^

Addressing this diagnostic gap, we develop and demonstrate a technique to image the deformation mechanics of microscale polymeric metamaterials during mechanical loading by using confocal microscopy. By capturing fluorescence from the polymerized material, we can produce high-fidelity three-dimensional renderings of these structures.

Furthermore, we capture a series of 3D images across discrete deformation steps to validate this technique. In the process, we show the ability to resolve individual fracture and buckling events inside the lattice, thereby contributing significantly to the precise assessment of their mechanical behavior.

One of the most common approaches for designing mechanical metamaterials is by constructing large lattice arrays composed of microscale unit cells. These are made with either repetitive unit cells or by patterning distinct unit cell states. We present a prototypical 4 × 4 × 4 lattice structure composed of four distinct unit cell states in Figure 1 that embodies this latter design rationale and serves as a basis for our proceeding investigations.

**Figure 1:**
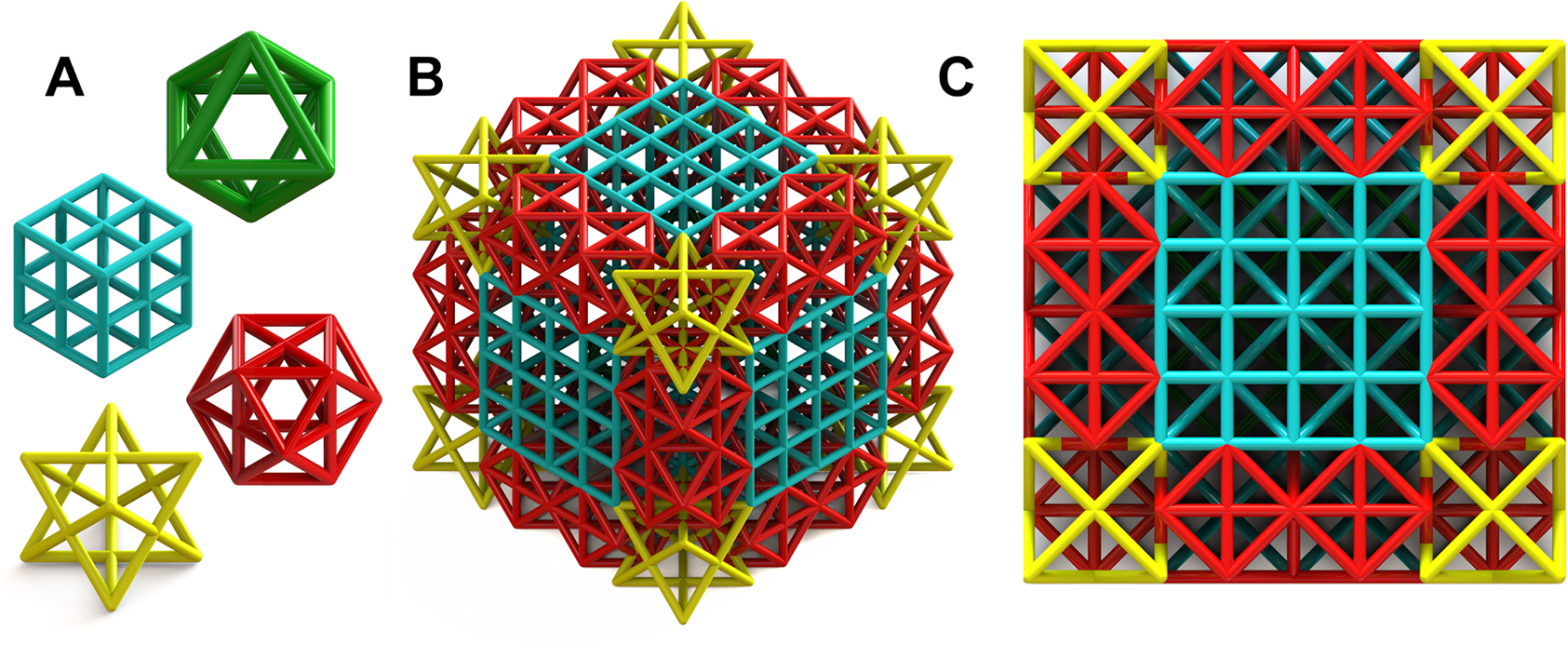
Lattice structure design. (A) Four constituent unit cells that are (B) patterned into a 4 × 4 × 4 arbitrary, symmetric lattice. Given the complex, inhomogeneous structure of this lattice it is difficult to view the internal members even from a (C) top view of the lattice. This engenders a need for more alternative imaging techniques to resolve these members and understand their deformation during mechanical loading.

We fabricate several copies of an 80 μm × 80 μm × 80 μm version of the same lattice design presented in Figure 1 by using a customized TPP setup. Details on this setup are discussed elsewhere in literature.^23^ SEM images of these structures are shown in Figure 2. Details about the resin materials and fabrication setup are provided in the Materials and Methods section.

**Figure 2:**
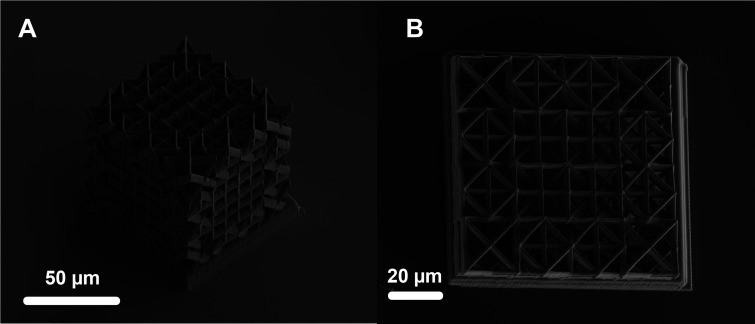
SEM Images. (A) Orthogonal view of the lattice structure design presented in Figure 1 that is fabricated with a two-photon polymerization setup. (B) Top view of the lattice shows internal beam members, but large sections of the view are masked by overlapping sections of the upper levels of the structure, making analysis of internal beam bending nearly impossible with these techniques.

Fabricated samples closely agree with the target geometry, with the elongated beam thicknesses originating from constraints associated with the voxel size from fabrication.^29^ We produce voxels with lateral dimensions ranging from 650 to 900 nm in size and axial dimensions spanning 3.2–4.0 μm. According to Zhou et al., the voxel resolution of TPP is approximately constrained as follows:^29^
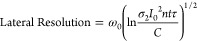
1
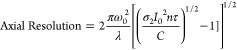
2where:
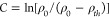


ω_0_ = beam waist

σ_2_ = effective two-photon cross section for the generation of radicals

*I*_0_ = the photon flux intensity at the beam center *r* = 0, *z* = 0

ρ_0_ = the primary initiator particle density

ρ_*th*_ = radical density threshold

τ = pulse width

n = number of pulses

t = total processing-irradiation time

Using an oil immersion lens, the beam waist can be described by the equation:
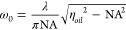
3

η_*oil*_ = refractive index of the oil medium

NA is the numerical aperture of the objective lens.

In practice, conventional TPP processes can achieve resolutions in the range 100–200 nm in the *xy*-plane and below 500 nm in the *z* axis. However, advanced techniques like stimulated emission depletion (STED) lithography have been successful in pushing these boundaries, achieving feature resolutions down to the tens of nanometers scale.^30^

In the effort to image the interior of the structures and especially to conduct this imaging simultaneous to compression testing, we first develop a method for reconstructing a three-dimensional representation of the lattice using confocal microscopy. According to the Rayleigh criterion, the resolution of a conventional confocal microscope is roughly constrained by^31,32^
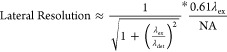
4
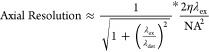
5where η is the refractive index of the medium. λ_ex_ is the excitation wavelength, and λ_det_ is the detected wavelength.

In practice, conventional confocal imaging yields lateral resolutions on the order of 200–600 nm and axial resolutions of 500–1000 nm with high numerical aperture oil objectives.^33^ These bounds are roughly in line with the capabilities of conventional TPP. In relation to the imaging of metamaterial structures, this would suggest that diffraction would limit the minimum spacing between disparate beam members rather than the minimum size of a structure that would be visible.

In our investigations, we utilize a 60× oil immersion objective with a numerical aperture of 1.25 to capture the features of our lattice structures. These images yield lateral dimensions of roughly 1–1.3 μm and axial dimensions of 3.8–4.5 μm which is larger than the dimensions measured by the SEM. Assuming that the beam sizes across the entire metamaterial structure are relatively uniform, the individual component images could be processed to create 3D arrays that closely reflect the true feature sizes of the structure, as measured to a datum such as SEM images.

We observed that the majority of the collected fluorescence appears to originate from the residual photoinitiator 4,4′-Bis(diethylamino)benzophenone, which is a component of the TPP resin. The fluorescence spectrum of this molecule is characterized by Ladika et al.^34^ and has a peak emission centered around 550 nm. Our testing suggests that at common excitation wavelengths of 405 and 488 nm the photopolymers can be sufficiently excited to produce images with high (>10:1) background contrast ratios at 50–120 μW of excitation. Other strategies for imaging are likely possible such as functionalizing the surface of a clear photo resin with fluorescent particles or dyes.

The refractive index of the polymerized resin material was measured to be approximately 1.48 at a wavelength of 488 nm. This agrees with similar measurements of the same polymer.^35^ Given that the refractive index mismatch of the polymer and surrounding air is large and that the lattice has a complex spatial variation in refractive index, scattering becomes dominant after imaging only a few layers into the structure, limiting the achievable imaging depth. To overcome this, we envelop the structures in a droplet of mineral oil whose refractive index was selected to match that of the photopolymerized resin to mitigate scattering from the structure, thus significantly reducing scattering and enhancing the imaging depth. In general, it is expected that this technique will be applicable to other photopolymerizable resin structures assuming that the resin is largely transparent and is either autofluorescent or functionalized with fluorescent particles or dyes. For best results, these structures should be immersed in fluids whose refractive index closely matches that of the resin.

Subsequently, cross-sectional images of the structure are taken in 100 nm increments to generate a stack of images for three-dimensional reconstruction. Select and distinct confocal microscope images of the fabricated structure are directly compared to 800 nm thick slices of their solid model counterparts in Figure S1 which demonstrate the ability of this technique to adequately capture the internal features of the structure at various depths. Furthermore, we develop an open-source code that binarizes the outputs of the individual confocal images to generate a point cloud to represent the structure and then subsequently generates a standard three-dimensional output file (.obj) of the structure. A simplified diagram of the imaging processing pipeline is shown in Figure S2.

Renderings of the lattice structure are shown in Figure 3. Orthogonal (Figure 3A) and top (Figure 3B) motifs closely resemble their solid model and SEM imaging counterparts. Notably, the axial beam thickness can be varied based on the method of imaging thresholding and binarization used during reconstruction and the lateral dimensions can be adjusted via dilation. Care should be taken to ensure that an appropriate processing methodology is used to produce reasonably accurate feature sizes.

**Figure 3:**
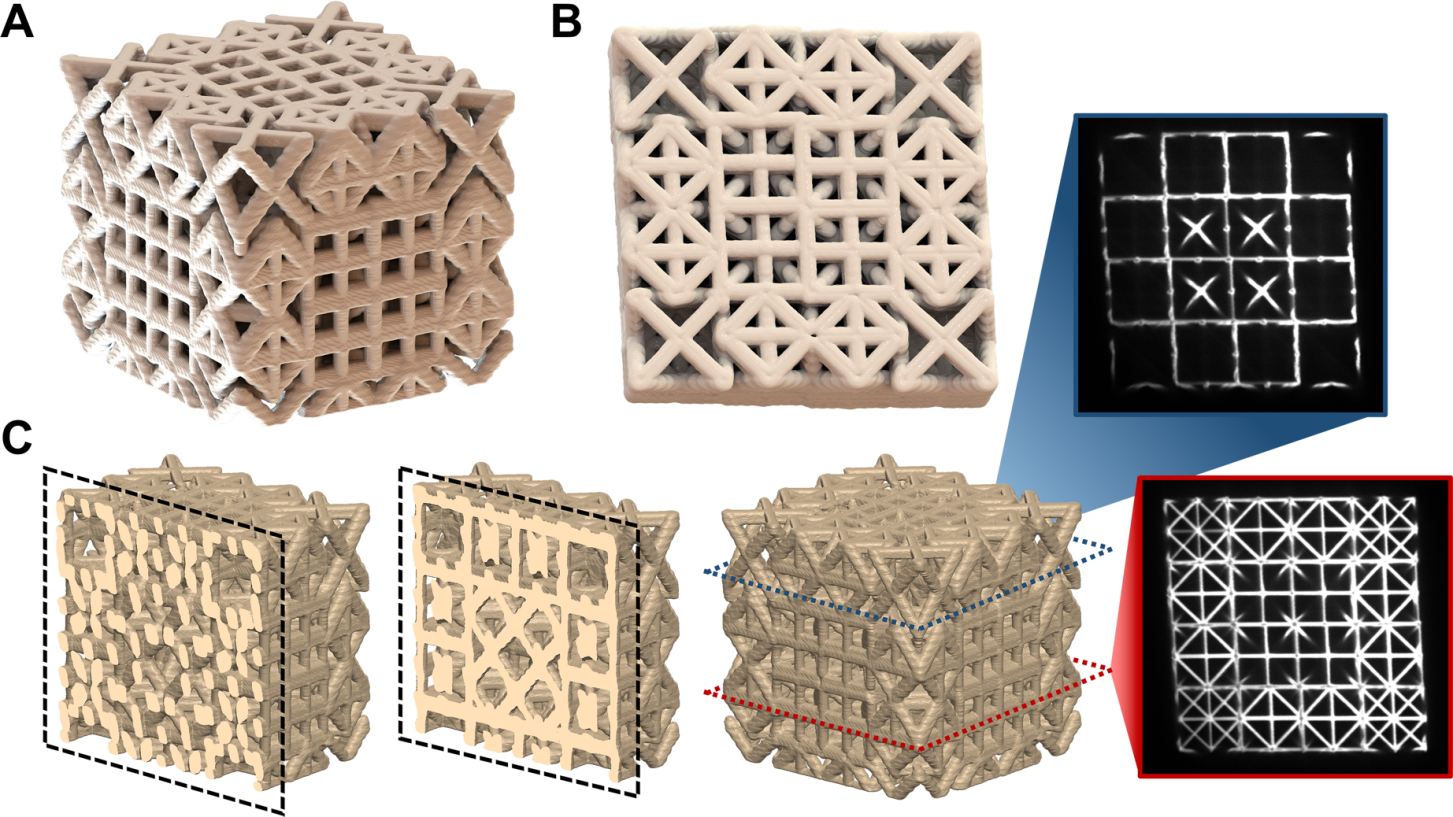
3D Renderings. (A) Orthogonal view of the lattice structure shown in Figure 2 that is imaged via confocal microscopy and digitally rendered. (B) Top view of the rendered structure. (C) (Left) Vertical sectional views depicting internal beam structure at two locations and (Right) Horizontal cross sections depicting the corresponding confocal image.

The cross-sectional views in Figure 3C clearly discern void spaces and the arrangement of beams inside the lattice. Beyond providing a three-dimensional visual representation, series of these reconstructions during mechanical compression can provide a basis to understand the deformation behavior of these structures—even deep inside the structure. This series of reconstructions, particularly during compression, grants insights into deformation behaviors that are imperceptible to SEM and HIM.

As a demonstration, we performed a mechanical compression test on the structure at various steps between 0 and 16 μm. We first construct a specialized *in situ* confocal micro compression apparatus shown in Figure 4 to incrementally compress structures and image between increments. Further details on this apparatus are included in the Materials and Methods section.

**Figure 4:**
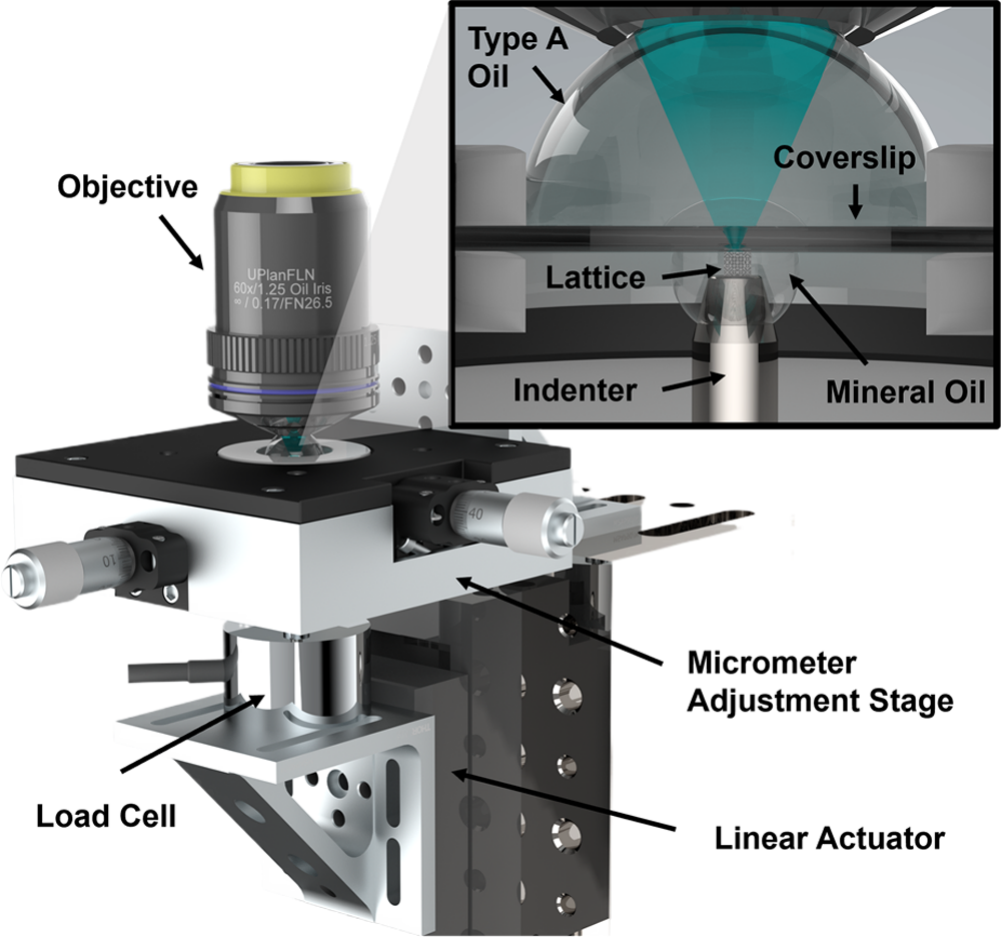
Compression apparatus and load curves. (A) Rendering of the microcompression apparatus used to study the metamaterials under loading conditions while simultaneously being imaged.

Our methodology involves successively imaging entire z-stacks that encompass the volume of the metamaterial structure before incrementally moving the indenter in 2 μm steps. Load curves from this experiment shown in Figure S3 discern loading events and highlight polymer relaxation during imaging. We should note that the incremental nature of the compression protocol does not generate a standard rate-controlled compression loading profile and thus measures periods of relaxation during imaging, wherein the structure is held at static intervals of loading. For measuring the effective Young’s modulus of a metamaterial, it may be necessary to perform rate-controlled compression tests avoiding incremental confocal imaging.

Renderings of the structure during loading increments of 2 μm, 6 μm, and 16 μm, as well as after releasing the indenter tip, are shown in Figure 5. These renderings show a clear progression of structure deformation where the beams in the upper layers first begin buckling at 2 μm (2.5% strain) of compression, before fracturing around 6 μm (7.5% strain) compression. A close look internally inside of the structure shows a chiral twisting of beams in the innermost unit cell before crumpling in proceeding loading events. This deformation is mostly confined to the second layer of unit cells from the top, and the crumple zone is best observed at the point of maximum compression (16 μm corresponding to ∼20% strain).

**Figure 5:**
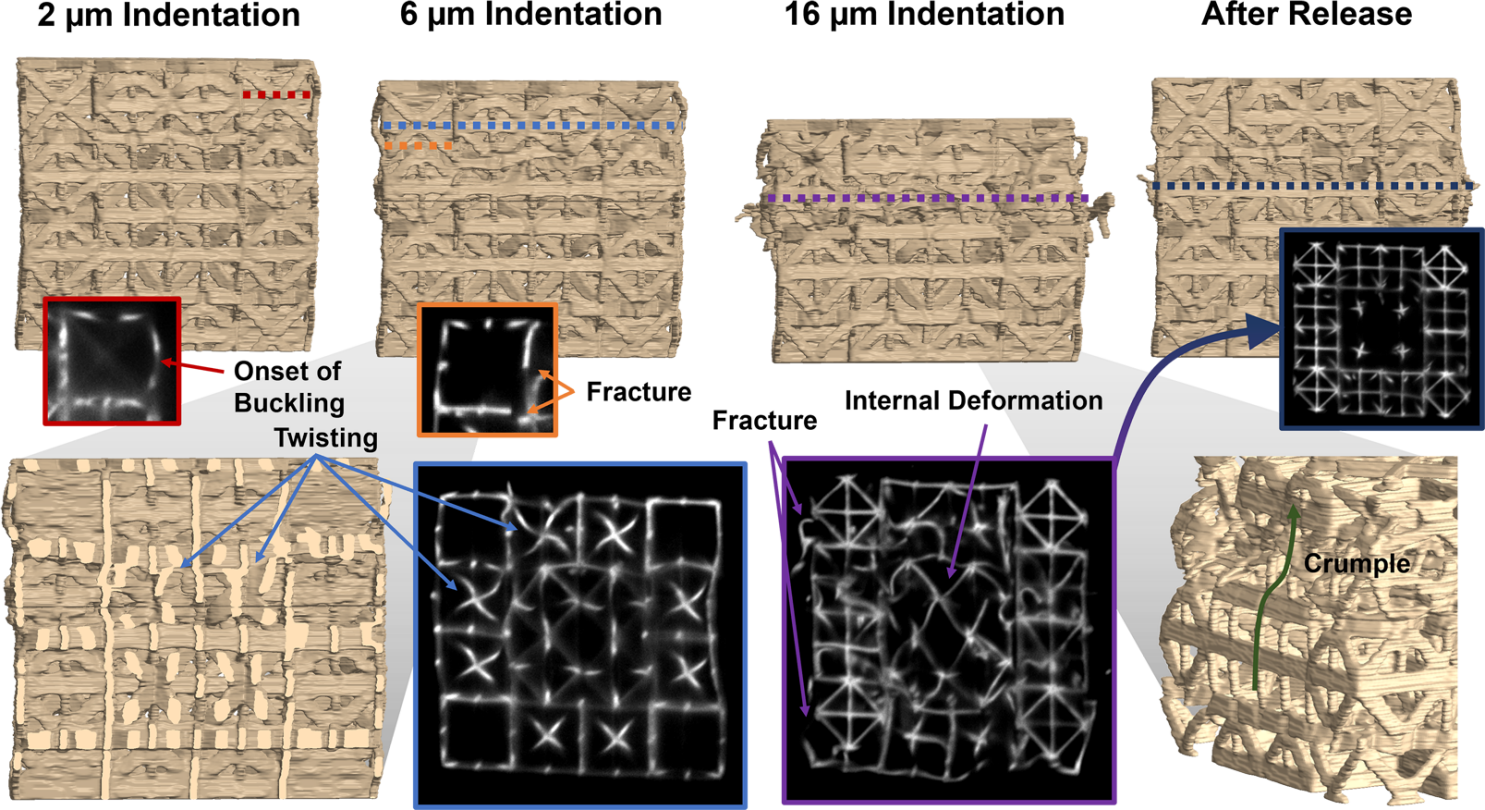
*In-situ* compression testing. (Top) 3D renderings at various indentation steps from 2 μm (left) to 16 μm (middle right) and after releasing the indentation tip (right). Various mechanical deformations across the different loading conditions are highlighted such as the onset of buckling, and the presence of fractures and plastic strain. (Bottom) (left) vertical cross-sectional view of the rendering depicting the beams twisting at 6 μm of compression. This same phenomenon is captured in a corresponding confocal slice (middle left, blue). At higher strains, sections of the metamaterial appear to crumple, which can be observed via imaging the internal structure (middle right) and investigating the exterior deformation of the structure (right).

Upon release of the indenter, the structure decompresses freely. Areas that experienced intense deformation recover to their original geometry, with only slight residual plastic deformation. This resilience is exemplified by a specific cross-section (highlighted in purple) that, while appearing significantly crumpled at the 16 μm deformation mark, retains minimal permanent deformation postunloading (indicated in dark blue). However, individual fractures across the volume of the structure are still visible after unloading, allowing a postexperimental failure analysis.

These experiments serve as a demonstration of the utility of using confocal microscopy techniques to analyze the deformation mechanics of microscale metamaterials with feature sizes less than one micrometer. The outcomes of these studies show that it is possible to capture individual buckling and fracture events across complex lattice structures that are commonly used in the design of mechanical metamaterials. Herein, we observe that the twisting of the internal unit cells seen at 6 μm of compression explains the onset of localized crumpling across the structure at subsequent stages of compression. These insights provide valuable information and a deeper understanding of how these structures internally deform, shedding light on the overarching deformation behaviors of such materials. In many cases, direct observation of internal deformation modes of complex architected materials as well as the onset of cracks across internal beam members and joints cannot be gained *in situ* with conventional techniques such as SEM or HIM.

Under extreme compression, the resolution of certain regions in our three-dimensional reconstructions becomes compromised as beam spacings fall beneath the microscope’s resolution capabilities and beams start to overlap in an unpredictable manner. Despite this limitation, sequential imaging during progressive compression stages allows us to put together an understanding of the deformation of beam elements under these conditions.

As we naturally seek to scale these techniques to larger materials with similar, submicrometer feature sizes, it becomes important to recognize the necessary trade-offs in imaging field of view and resolution required. Generally, higher numerical aperture lenses which can resolve smaller feature sizes have smaller working distances, which limit the maximum height that can be observed. Drawing inspiration from recent breakthroughs in mesoscale biological imaging could inform strategies to balance these trade-offs, enabling the study of larger systems.^36,37^

While our experiments focus on the deformation of lattices, the same tools can also be used to study biological and hybrid systems. Recent work has demonstrated cellular scaffolds of desired shapes and sizes that can be populated by cells.^38−41^ Labeling the cells with spectrally distinct fluorophores from the TPP structures would enable the simultaneous imaging of both the scaffolding structure and the cells and how they interact during the tissue generation process.^42,43^ For example, substantial active forces and deformations have been observed in 3D human microtissue models on metamaterial scaffolds.^44^

Our study presents a robust technique for high-resolution optical imaging of microscale metamaterials using confocal microscopy. Through our investigation of a prototypical structure measuring 80 μm × 80 μm × 80 μm, we have demonstrated the capability of this method to capture and quantify internal deformations and fractures under a mechanical load with remarkable precision. Notably, the ability to 3D visualize the progressive deformation of lattices at such fine scales is a leap forward, overcoming the limitations of traditional imaging modalities. The success of this technique in characterizing the behavior of microscale metamaterials under stress opens new avenues for its application. Beyond enabling a more insightful analysis of mechanical metamaterials, there is potential for this technique to be utilized for imaging the deformation of cellular scaffolding to understand how different biological systems respond to imposed and self-induced stresses in their environments.

## Materials and Methods

A hybrid organic–inorganic resin, SZ2080 is used with Zr-DMAEMA (30 wt %) as a binder. The resin is composed of 70 wt % zirconium propoxide and 10 wt % (2-dimethylaminoethyl) methacrylate (DMAEMA) (Sigma-Aldrich). See Ovsianikov et al. for more information.^45^

Structures were fabricated using submicrometer resolution direct femtosecond laser writing using two-photon polymerization on SZ2080 photoresist. The setup uses a FemtoFiber Pro NIR laser, which emits 780 nm, 100 fs fwhm, pulses at 80 MHz. Polymerization of the resin is achieved with a 1.3 NA microscope objective lens (Plan-Apochromat 40 × /1.3 Oil Olympus). The laser output energy was measured before the objective lens at 6 mW. The resin sample is positioned with three axis piezo and servo stages.

We utilized a Bruker Swept-Field Confocal microscope for imaging using an excitation wavelength of 488 nm and using a 488 nm long pass filter. Images were generated of the structure at pixel resolutions of 512 × 512- and 16-bit intensity resolution. Attached to the microscope stage is our custom compression setup seen in Figure 4 that enables movement of metamaterial structures relative to the indentation tip, and the entire compression apparatus relative to the microscope objective. The indentation tip has a relatively flat (1°) angled square tip that is approximately 200 μm across that is mounted to a 0.1N load cell (Novatech F329).

The imaging sequence begins by first imaging the entire structure in 100 nm intervals with layer spacing. After the entire structure was imaged, the compression apparatus would apply a user defined strain. In our studies, the apparatus moved 2 μm increments. During this time, images were not taken. Afterward the whole structure would be imaged while remaining at constant applied strain, before compressing the structure further. This cycle repeats until the compression apparatus is released, and the postcompressed structure is image. We demonstrate the ability to image at rates exceeding 4 2D slices per second, although faster rates are possible.

## Data Availability

Our code for rendering the confocal images is made publicly available at: https://github.com/naichenzhao/Confocal-Rendering/tree/main?fbclid=IwAR0MIdlTX0NbsaCvXUi0f1v7Nk4WsUEDBFAkT7gWLbY_CdAXAwpV7Z0MnnA

## Supporting Information Available

CAD cross-sectional slices compared to confocal images, diagram of the image processing procedure, load curves from compression tests, axial line width measurements. (PDF)
