# Supplementary material for: Three-Dimensional Optical Imaging of Internal Deformations in Polymeric Microscale Mechanical Metamaterials
Source: Nano Lett. 2024 Jan 26;24(9):2735–42. doi: 10.1021/acs.nanolett.3c04421 (PMC10921468; doi:10.1021/acs.nanolett.3c04421)
Supplement: Supplementary file 1 — nl3c04421_si_001.pdf [file nl3c04421_si_001.pdf]

Supporting Information for: Three-Dimensional Optical Imaging of Internal Deformations in Polymeric Microscale Mechanical Metamaterials

Brian W. Blankenship[1†], Timon Meier[1†], Naichen Zhao[1†], Stefanos Mavrikos[1], Sophia Arvin[1], Natalia De La Torre[1], Brian Hsu[1], Nathan Seymour[1], Costas P. Grigoropoulos[1*]

1. Laser Thermal Laboratory, Department of Mechanical Engineering, University of California, Berkeley, CA 94720, USA

† These Authors contributed equally
*Corresponding Author

Email: cgrigoro@berkeley.edu

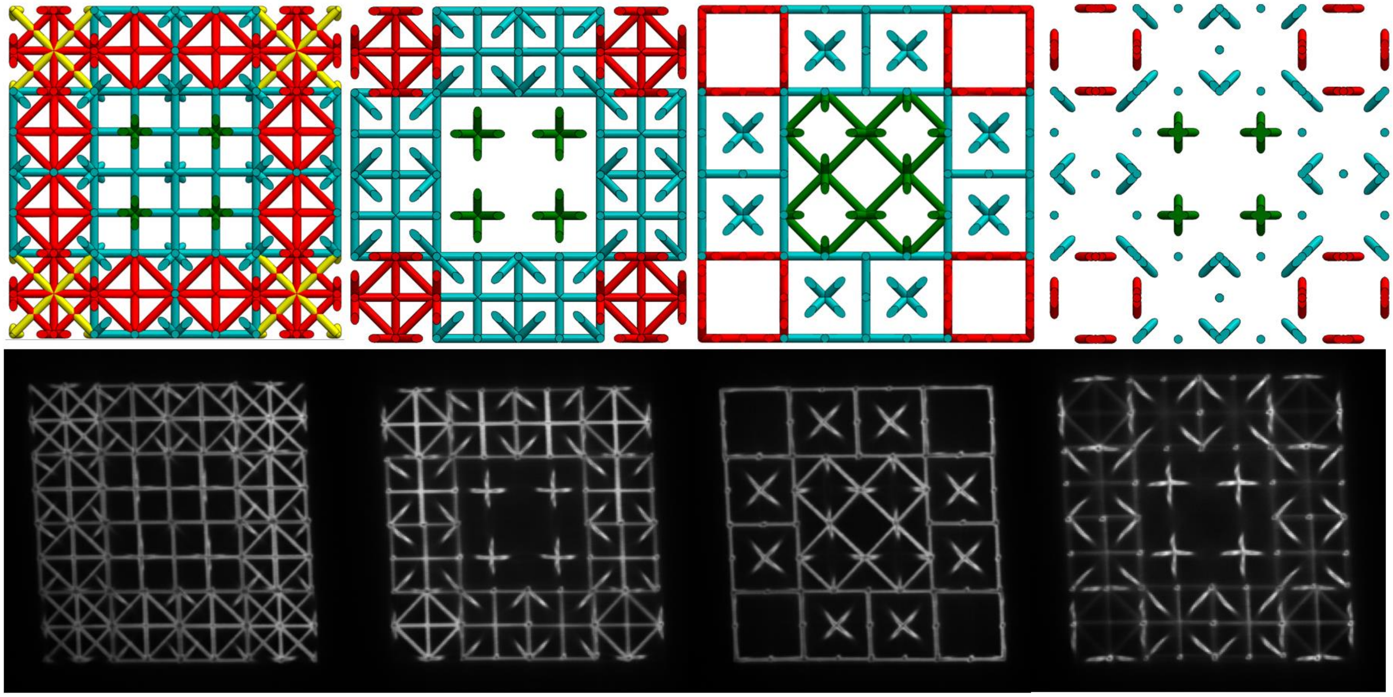

Figure S1: Confocal Images (Top) CAD renderings of 800 nm thick slices of the 4 x 4 x 4 lattice structure. (Bottom) Corresponding confocal images showing remarkable resemblance to the ideal shape.

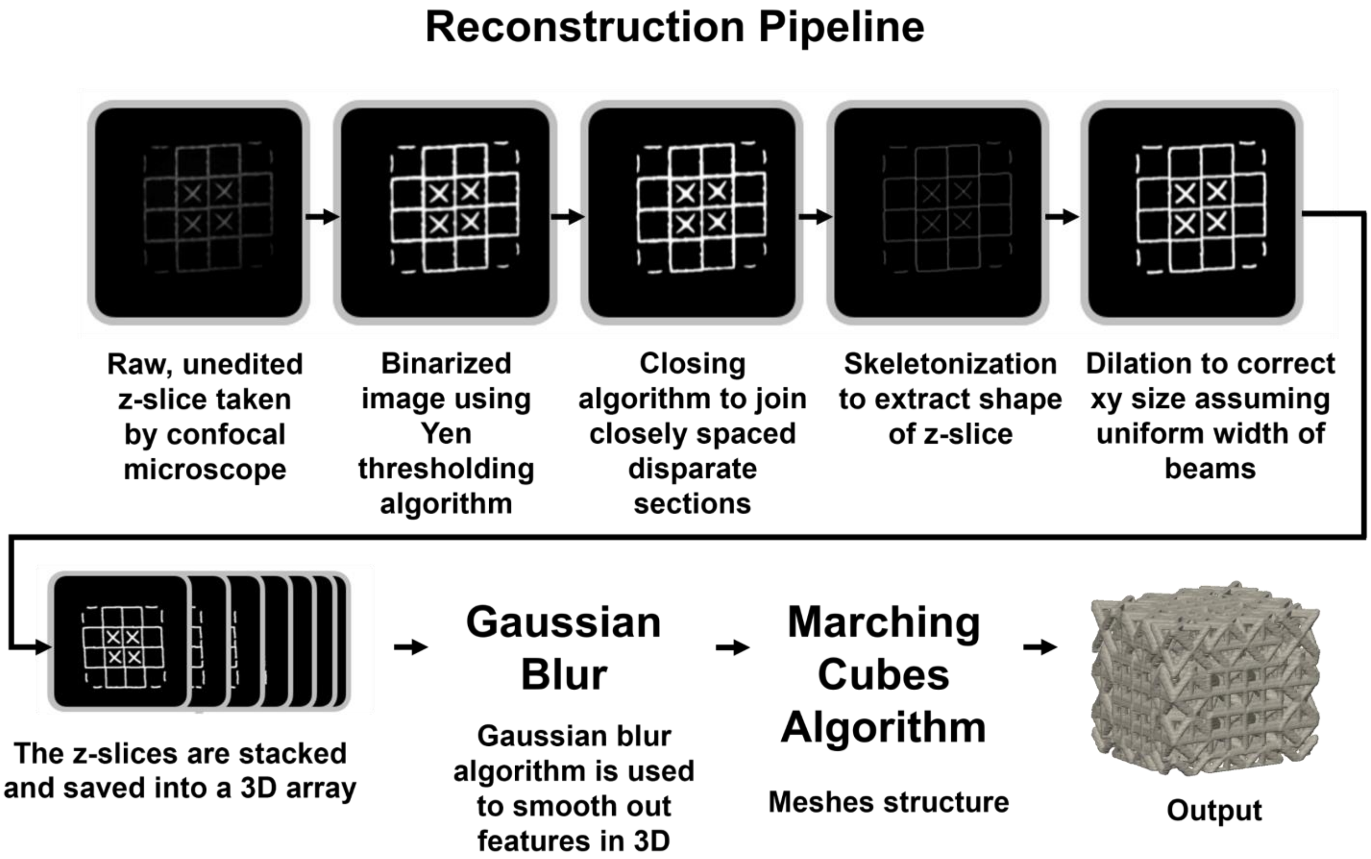

Figure S2: Image Processing Pipeline Generalized diagram for how confocal images are processed and how 3D structures are generated with stacks of images. Z-Slices, which are taken in user defined increments are first thresholded using the Yen thresholding algorithm.[1] Subsequently, images are closed and skeletonized before being dilated to approximately correct xy sizes via comparison to SEM images. Afterwards the binarized slices are stacked and saved in a 3D array which is smoothed, and then converted to an appropriate file format.

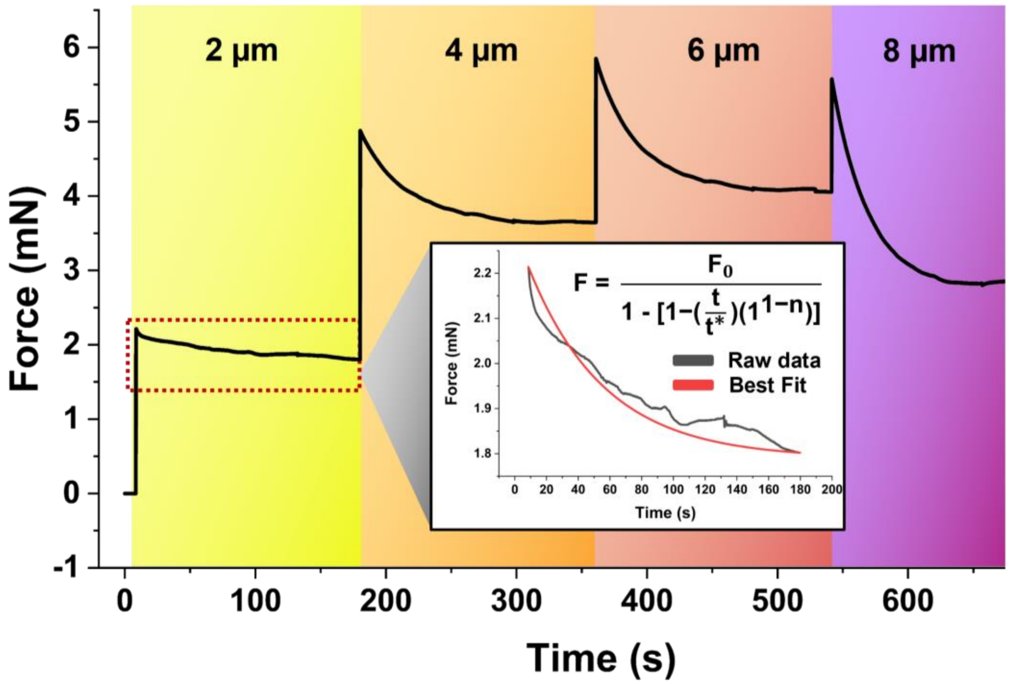

Figure S3: Load Curves The structures were compressed in increments of 2 µm. The indenter tip was set to remain static over the course of imaging before successive indentation steps. Because of this waiting period, the load cell captures the relaxation from the polymer. This relaxation curve is compared to a best fit of the Obukhov stress relaxation model shown above. [2]

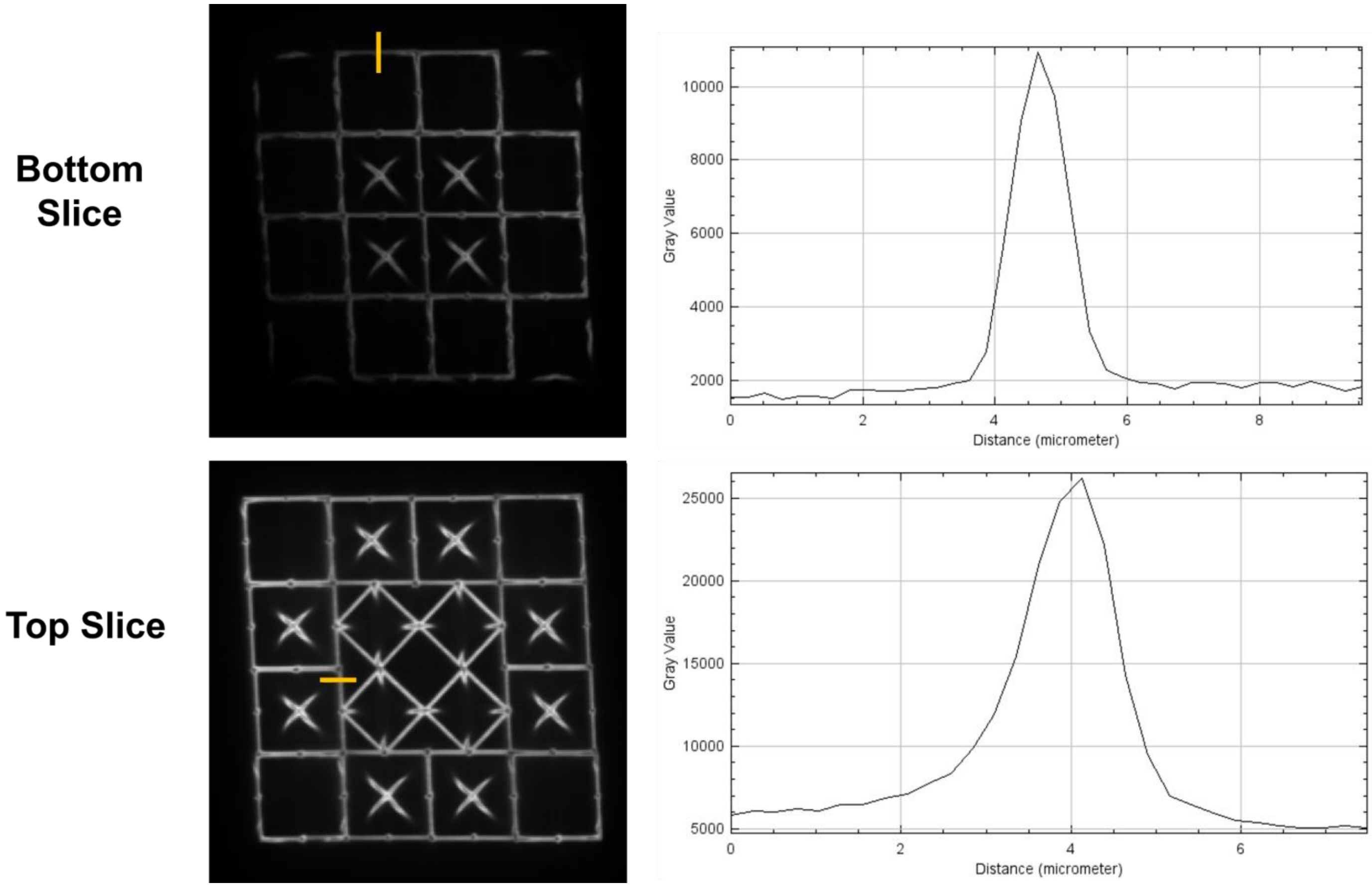

Figure S4: Linewidth measurements Intensity profiles (yellow) of beam members as measured from the confocal images taken of the metamaterials from the top slice (where the passes the furthest through the structure) and the bottom slice (where the laser travels the least through the structure) are shown. Across the entire structure, the lateral FWHM of the beam members tends to vary between 1- 1.3 µm.

References

(1) Yen, J. C.; Chang, F. J.; Chang, S. A New Criterion for Automatic Multilevel Thresholding. *IEEE Trans Image Process* **1995**, *4* (3), 370–378. https://doi.org/10.1109/83.366472.

(2) Junisbekov, T. M.; Kestel′man, V. N.; Malinin, N. I. *Stress Relaxation in Viscoelastic Materials*; Science Publishers, 2003.
